# Exosomes from human urine-derived stem cells in porcine urethral scaffold construction

**DOI:** 10.1515/biol-2025-1320

**Published:** 2026-05-04

**Authors:** Kai-Yue Zhang, Hao Zhong, Wei-De Ma, Xiao-Yan Yang, Li-Zhong Han, Zhi-Zhong Liu

**Affiliations:** Department of Urology, Inner Mongolia Baogang Hospital, Baotou, 014010, China

**Keywords:** decellularized matrix, exosome, tissue engineering, urethral stricture, urine-derived stem cells

## Abstract

This study aims to examine the role of exosomes derived from human urine-derived stem cells (hUSCs-Exo) in the *in vitro* construction of a composite structure comprising hUSCs and a porcine urethral decellularized matrix. hUSCs-derived exosomes were isolated and characterized, and hUSCs were treated with varying exosome concentrations to assess migration using scratch and Transwell assays. Dio-labeled hUSCs were seeded onto porcine urethral decellularized matrices and grouped by exosome concentration (0, 50, 100 μg/mL). Cell proliferation and distribution were examined under a fluorescence inverted microscope on days 1, 3, and 7. On day 7, samples were paraffin-embedded for histological analysis of cell integration. hUSCs with mesenchymal stem cell (MSC) properties were successfully isolated, and exosomes extracted via centrifugation. hUSCs-exosome (Exo) enhanced cell migration but did not significantly affect proliferation. Dio-labeling and H&E staining confirmed hUSC presence and attachment to the urethral matrix, while CD44 immunohistochemistry confirmed the presence and attachment of hUSCs within the scaffold. Exosomes derived from hUSCs did not significantly enhance cell proliferation in the construction of the porcine urethral decellularized matrix-hUSC complex. The specific exosomal cargo responsible for these differential effects on migration versus proliferation was not examined in this study and will be the focus of future investigations.

## 1 Introduction

Urethral stricture is a common condition in urology. Short-segment strictures (<2 cm) can usually be effectively with endoscopic interventions or urethral anastomosis, in contrast, long-segment strictures (≥2 cm) remain a major therapeutic challenge [1]. Current treatments for long-segment urethral strictures mainly rely on involve urethral reconstruction using autologous tissue grafts, such as buccal mucosa, foreskin, or skin flaps. However, these methods are associated with relatively high rates of urinary incontinence and stricture recurrence [2]. In addition, harvesting autologous grafts casues secondary tissue injury and increases the risk of bleeding, infection, and other postoperative complications [3].

Advances in regenerative medicine and tissue engineering have prompted the investigation of various scaffold materials for urethral repair, including synthetic polymers, collagen-based materials, and decellularized matrices [4]. However, the use of scaffold alone is limited by the size of the defect. When the urethral defect exceeds 0.5 cm, cell migration across the entire defect becomes insufficient, increasing the risk of postoperative fibrosis and restenosis [5].

Stem cells exhibit strong proliferative capacity and multilineage differentiation potential. They also possess immunomodulatory properties that regulate inflammatory responses, promote tissue remodeling, and support extracellular matrix organization within the local microenvironment [6]. Human urine-derived stem cells (hUSCs) are a novel type of mesenchymal stem cells that can be noninvasively isolated from voided urine. This method is cost-effective and painless for patients [7]. Previous studies have demonstrated the potential of hUSCs in multiple tissue repair applications [8], [9], [10]. Therefore, hUSCs represent a promising cell source for urethral tissue engineering, particularly when combined with suitable scaffold material.

Exosomes are extracellular vesicles with diameters of approximately 30–150 nm that are released by cells through exocytosis. They can be internalized by recipient cells and mediate intercellular communication by transferring bioactive molecules locally and systemically. Increasing evidence indicates that exosomes participate in a wide range of physiological and pathological processes. They are secreted by many cell types and play important roles in skin wound healing, angiogenesis, and muscle repair. For instance, Shi et al. reported that locally administered bone marrow mesenchymal stem cell–derived exosomes reduced urethral stricture formation by promoting angiogenesis and inhibiting fibrosis [11]. Similarly, Chen et al. demonstrated that stem cell–derived exosomes significantly attenuated stricture severity and collagen deposition in a rabbit model of post-traumatic urethral stricture, mainly through inhibition of Interleukin-1β [12]. These findings highlight the therapeutic potential of exosomes in tissue repair and regeneration.

Accordingly, this study aims to construct a porcine urethral decellularized matrix fiber scaffold co-cultured with hUSCs. Three experimental groups are established and treated with different concentration of hUSC-derived exosomes (hUSCs-Exo) for one week. Cell proliferation and integration will be assessed using fluorescence inverted microscopy, hematoxylin and eosin staining, and immunohistochemical analysis to assess the role of hUSCs-Exo in composite scaffold formation.

## 2 Materials and methods

### 2.1 Culture and passage of urine-derived stem cells

#### 2.1.1 Collection of sterile urine from healthy adults

In this experiment, fresh urine samples were collected from healthy adult volunteers aged 20–30 years. Prior to sample collection, volunteers disinfected the external urethral orifice using 70 % ethanol. A volume of 200 mL midstream urine was obtained from each participant, with care taken to prevent contamination of the bottle cap and the sample during the collection process.

**Informed consent:** Informed consent has been obtained from all individuals included in this study.

**Ethical approval:** The research related to human use has been complied with all the relevant national regulations, institutional policies and in accordance with the tenets of the Helsinki Declaration, and has been approved by the Ethics Committee of Inner Mongolia Baogang Hospital (No. 2021-MER-084).

#### 2.1.2 Extraction and culture of hUSCs

Collected urine samples were centrifuged at 1,500 r/min for 10 min at room temperature, and the supernatant was discarded. Four milliliters of complete hUSC culture medium were added, and the pellet was gently resuspended. The resulting cell suspension was seeded into a 12-well plates and incubated at 37 °C with a humidified atmosphere containing 5 % CO_2_. Cell adhesion was observed after 2 days, and the culture medium was replaced every 2 days. When adherent cells reached approximately 50–70 %, passaging was initiated, and the cells were designated as passage 0 (P0) hUSCs.

For the first passage, the culture medium was removed, and 0.25 % trypsin was added. Cells were incubated at 37 °C for 2 min to induce detachment. Cell morphology was monitored under an optical microscope, and digestion was terminated by adding complete hUSC culture medium once cell shrinkage and rounding were observed. The plate was gently tapped under a laminar flow hood, and the cells were pipetted to obtain a single-cell suspension. This suspension was centrifuged at 1,200 r/min for 5 min at room temperature, after which the supernatant was discarded. The cell pellet was resuspended in complete culture medium and seeded into 6-well plates as passage 1 (P1) hUSCs.

Cells were maintained at 37 °C with 5 % CO_2_, and the medium was replaced every 2 days. When approximately 70 % confluence was reached, a second passage was performed. The digested and centrifuged cells were resuspended in complete hUSC culture medium, transferred to a T25 culture flasks, and supplemented with additional medium to generate passage 2 (P2) hUSCs. Medium changes were performed every 2 days. When cells reached approximately 90 % confluence, further passaging was conducted at a split ratio of 1:2 or 1:3. hUSCs were expanded to passage 2–3 at 70–90 % confluence before use; seeding densities were 2 × 10^4^ cells/mL for differentiation studies and approximately 1 × 10^5^ cells per scaffold (200 µL suspension). Exosomes were quantified by protein concentration (50 and 100 μg/mL) as determined by BCA assay.

### 2.2 Identification of hUSCs

#### 2.2.1 Flow cytometry detection of surface markers on hUSCs

Third-generation hUSCs exhibiting a confluence rate exceeding 90 % were collected and subjected to digestion with 0.25 % trypsin, followed by centrifugation at 1,200 r/min for 5 min at room temperature. After discarding the supernatant, the cell pellet was resuspended in phosphate-buffered saline (PBS). A 200 μL aliquot of the cell suspension was transferred into EP tubes, and monoclonal antibodies against cluster of differentiation (CD) markers CD105, CD44, CD34, CD90, CD73, and human leukocyte antigen-DR (HLA-DR) were added to individual tubes. The samples were incubated in the dark at room temperature for 30 min and subsequently analyzed using flow cytometry.

#### 2.2.2 Induced differentiation of hUSCs

##### 2.2.2.1 Adipogenic induction

Third-generation hUSCs were collected and adjusted to a cell concentration of 2 × 10^4^/mL. The cells were seeded into 6-well plates at 2 mL per well in complete hUSC culture medium. Once the cells reached full confluence, adipogenic differentiation was initiated by adding 2 mL of adipogenic induction medium solution A. Solution A consisted of 88.4 % Oricell basal medium, 10 % fetal bovine serum (FBS), 1.5 % human-related stem cell adipogenic differentiation supplement A-I, and 0.1 % supplement A-II. After 3 days, solution A was removed and replaced with 2 mL of adipogenic induction medium solution B, which contained 90 % Oricell basal medium, 10 % FBS, and 0.2 % human-related stem cell adipogenic differentiation supplement B. Solutions A and B were alternated throughout the induction period.

After 21 days adipogenic induction, cells were fixed with 2 mL of cell fixative per well at room temperature for 30 min. The fixative was then removed, and 2 mL of diluted Oil Red O staining solution supernatant was added to each well and incubated for 30 min at room temperature. Lipid droplet formation was evaluated under a light microscope.

##### 2.2.2.2 Osteogenic induction

Third-generation hUSCs were collected. Upon reaching 70 % confluence in the 6-well plate, 2 mL of osteogenic induction medium was added to each well, and the plate was placed in an incubator to initiate induction. Following a 3-week induction period, cell morphology was examined, and Alizarin Red staining solution was applied for 10 min at room temperature. The staining solution was then thoroughly rinsed off, and the stained cells were observed under a microscope.

##### 2.2.2.3 Chondrogenic induction

Third-generation hUSCs were collected, and upon reaching 90 % confluence, the culture medium was replaced with chondrogenic induction medium. After a 3-week induction period, cell morphology was examined, and Alcian Blue staining solution was applied. The staining results were subsequently assessed under a microscope.

### 2.3 Extraction and identification of hUSCs-Exo

#### 2.3.1 Extraction of hUSCs-Exo

Once hUSCs reached full confluence, the culture medium was replaced with exosome-free serum medium. Cells were then cultured for an additional 48 h. The conditioned medium was collected and filtered through a 0.22 μm membrane. Exosome extraction reagent (15 mL) was added to the filtrate, followed by static incubation at 4 °C overnight. The mixture was subsequently centrifuged at 3,220×*g* for 60 min. After centrifugation, the supernatant was discarded, and the exosome pellet adhering to tube wall was retained. The exosomes were resuspended, transferred to 1.5 mL microcentrifuge tubes, and stored at −80 °C until further use.

#### 2.3.2 Identification of hUSCs-Exo

Transmission electron microscopy was used to observe the morphology and size of hUSCs-Exo. Exosome particle size and concentration were measured using nanoparticle tracking analysis (NTA).

#### 2.3.3 Protein extraction from hUSCs-Exo

Radio immunoprecipitation assay (RIPA) lysis buffer was thawed on ice, and phenylmethylsulfonyl fluoride (PMSF) was added at a ratio of 100:1 (RIPA:PMSF). Exosomes were thawed on ice and lysed for 10 min under the same conditions. Following lysis, the samples were centrifuged at 12,000×*g* for 5 min at 4 °C. The resulting supernatant was collected into EP tubes and stored in a −80 °C freezer for subsequent experimental use.

#### 2.3.4 Determination of protein concentration in hUSCs-Exo

A bicinchoninic acid (BCA) protein assay kit was used to quantify the protein concentration in hUSCs-Exo. Absorbance values for both samples and standards were measured at a wavelength of 562 nm using a microplate reader, and protein concentrations were calculated based on the corresponding standard curve.

#### 2.3.5 Nanoparticle tracking analysis of exosome particle size

Approximately 1 mg of sample was ultrasonically dispersed for 5 min. Measurements were conducted using a Malvern Zetasizer Nano ZS90. Each sample was measured in triplicate to ensure reproducibility.

### 2.4 Preparation of porcine urethral decellularized matrix

Urethras were obtained from five 6-month-old male pigs. The foreskin and penile corpus cavernosum were completely removed, and the urethras were cut into 2 cm segments. The segments were immersed in a 3 % hydrogen peroxide for 30 min, followed by treatment with 0.25 % trypsin and shaking digestion at 37 °C for 24 h. After digestion, the urethral segments were removed, disinfected, and rinsed with distilled water containing 1 % gentamicin-streptomycin. The samples were then stored at 4 °C until further use. Hematoxylin-eosin (H&E) staining was performed to evaluate the extent of urethral decellularization.

### 2.5 Cell scratch assay

Third-generation hUSCs were digested and seeded into three 6-well plates, then cultured under standard conditions. Once the cells reached full confluence, the culture medium was removed, and linear scratches were created using a 200 μL pipette tip to establish a scratch wound model. hUSCs-Exo were added at concentrations of 0, 50, and 100 μg/mL, with two replicate wells assigned per group. Images were captured immediately after scratching (0 h), and additional images were obtained at 12, 24, 36, and 48 h during incubation.

### 2.6 Transwell assay to detect the effect of hUSCs-Exo on hUSCs migration ability

Three Transwell chambers were placed into 6-well culture plates. One milliliter of hUSC suspension was added to the upper chamber of each insert, and hUSCs-Exo were applied at concentrations of 0, 50, and 100 μg/mL. One milliliter of low-serum medium was added to the lower chambers, and the plates were incubated for 24 h. After incubation, the inserts were transferred to new plates, washed with PBS, and fixed for 20 min. Cells that migrated to the lower surface of the membrane were stained with 0.1 % crystal violet for 15 min and counted under an inverted microscope.

### 2.7 Construction of porcine urethral decellularized matrix and hUSCs complex

#### 2.7.1 Dio staining of hUSCs

Second-generation hUSCs were collected and the original culture medium was removed. An appropriate volume of the green, fluorescent cell membrane dye (Dio) was added to the culture flask, which was then incubated at 37 °C for 20 min in the dark. Following incubation, the staining solution was removed, and the cells were washed three times with PBS. Subsequently, 5 mL of complete hUSC culture medium was added. The stained cells were observed using a fluorescence inverted microscope.

#### 2.7.2 Complexation of porcine urethral decellularized matrix with hUSCs

Porcine urethral decellularized matrices were retrieved from a −20 °C freezer and thawed at room temperature. The culture medium was replaced daily for three consecutive days. All procedures were performed under sterile conditions in a laminar flow hood. The matrices were laid flat in 24-well plates and preincubated in complete hUSC culture medium for 2 h. Tthe medium was then removed, and 200 μL of Dio-stained P2 hUSC suspension was added to each well. Plates were incubated at 37 °C, and an additional 200 μL of cell suspension was added after 4 h.

The hUSC-seeded matrices were divided into three groups and treated with hUSCs-Exo at protein concentrations of 0, 50, and 100 μg/mL. Each group included two replicate wells. After 1 day of culture, the urethral matrices were transferred to new 24-well plates, observed under a fluorescence inverted microscope, and imaged. Cultures were maintained with daily fluorescence microscopy to monitor cell growth. Images were captured on days 3 and 7, and cell numbers were quantified using the five-point sampling method for statistical analysis. The tissue constructs were subsequently embedded in paraffin blocks and examined via H&E staining.

#### 2.7.3 Immunohistochemical identification of hUSCs in the complex

Paraffin-embedded tissue constructs were sectioned into 4 μm slices using a microtome and baked for 1 h. Sections were deparaffinized in xylene and rehydrated through a graded ethanol series (100 %, 95 %, and 90 %). Antigen retrieval was performed by heating the sections in ethylene diamine tetraacetic acid (EDTA) buffer.

Endogenous peroxidase activity was blocked by incubating the sections with peroxidase blocking solution for 10 min at room temperature. Normal non-immune animal serum was applied to block nonspecific binding, and the sections were incubated in a humid chamber for 10 min. Primary antibody was then applied, and the sections were incubated overnight incubation at 4 °C. After washing with PBS, the appropriate secondary antibody was added and incubated for 10 min at room temperature, followed by incubation with streptavidin-peroxidase solution for an additional 10 min.

Color development was achieved using a chromogenic reagent for 7 min. The sections were then rinsed with distilled water and counterstained with hematoxylin for 1 min. Dehydration was performed through a graded ethanol series (85 %, 95 %, and two changes of 100 %), with each step lasting 2–3 min. Sections were cleared in xylene, air-dried, mounted, and examined under a light microscope.

### 2.8 Statistical analysis

SPSS 22.0 and GraphPad Prism were utilized for statistical analysis. Prior to *t*-tests, normality was assessed using the Shapiro–Wilk test and homogeneity of variances using Levene’s test; all data met these parametric assumptions. Mean cell counts were expressed as *x* ± *s*. Comparisons between groups were conducted using *t*-tests, multiple group comparisons using Kruskal Wallis test with a *p*-value of <0.05 considered statistically significant.

## 3 Results

### 3.1 Morphology of hUSCs

**Figure 1:**
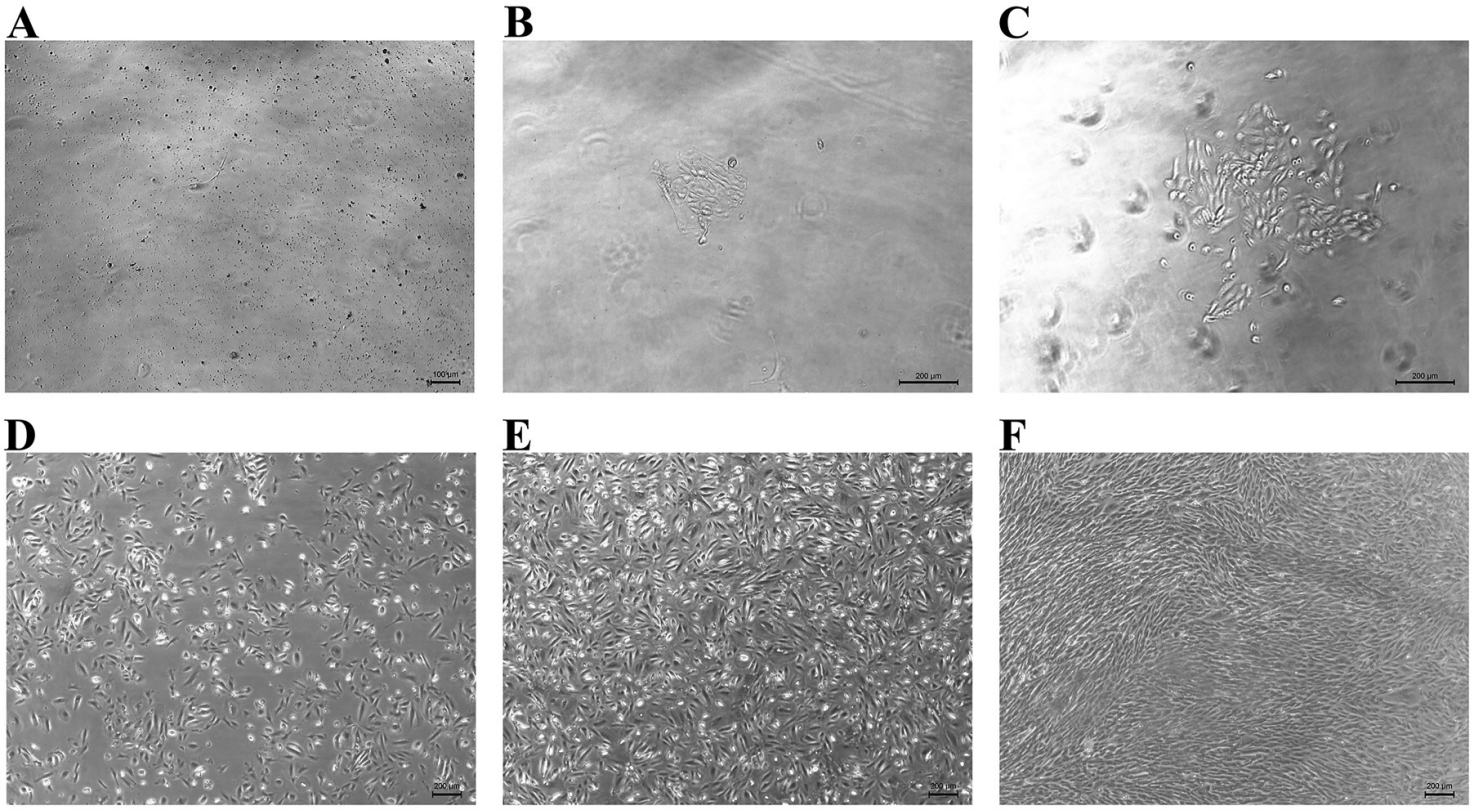
Morphological observation of hUSCs during *in vitro* culture. (A) Morphology of hUSCs at day 2 of culture (100×), with the arrow indicating a single hUSC. (B) Morphology at day 4 of culture (100×), indicating a cobblestone-like appearance. (C) Morphology at day 10 of culture (100×). (D) morphology at day 14 of culture (50×), with evidence of accelerated proliferation. (E) morphology prior to passage 0 (P0) (50×), indicating approximately 70 % confluence. (F) Morphology prior to passage 3 (P3) (50×).

Scattered adherent cells were observed 2 days after culture initiation. By day 4, the adherent cells formed colonies with a cobblestone-like morphology. Around day 10, these colonies began to proliferate, and by day 14, rapid cell expansion was evident, with cells exhibiting a rice grain-like appearance. By approximately day 16, cell confluence in 12-well plates reached 50–67 %. After passaging into T25 culture flasks, the proliferation rate increased markedly, with confluence exceeding 90 % within 4–5 days, at which point further passaging was performed. As proliferation progressed, cell morphology gradually transitioned to a spindle-shaped or fusiform appearance (Figure 1).

### 3.2 Flow cytometer detection of surface markers for hUSCs identification

**Figure 2:**
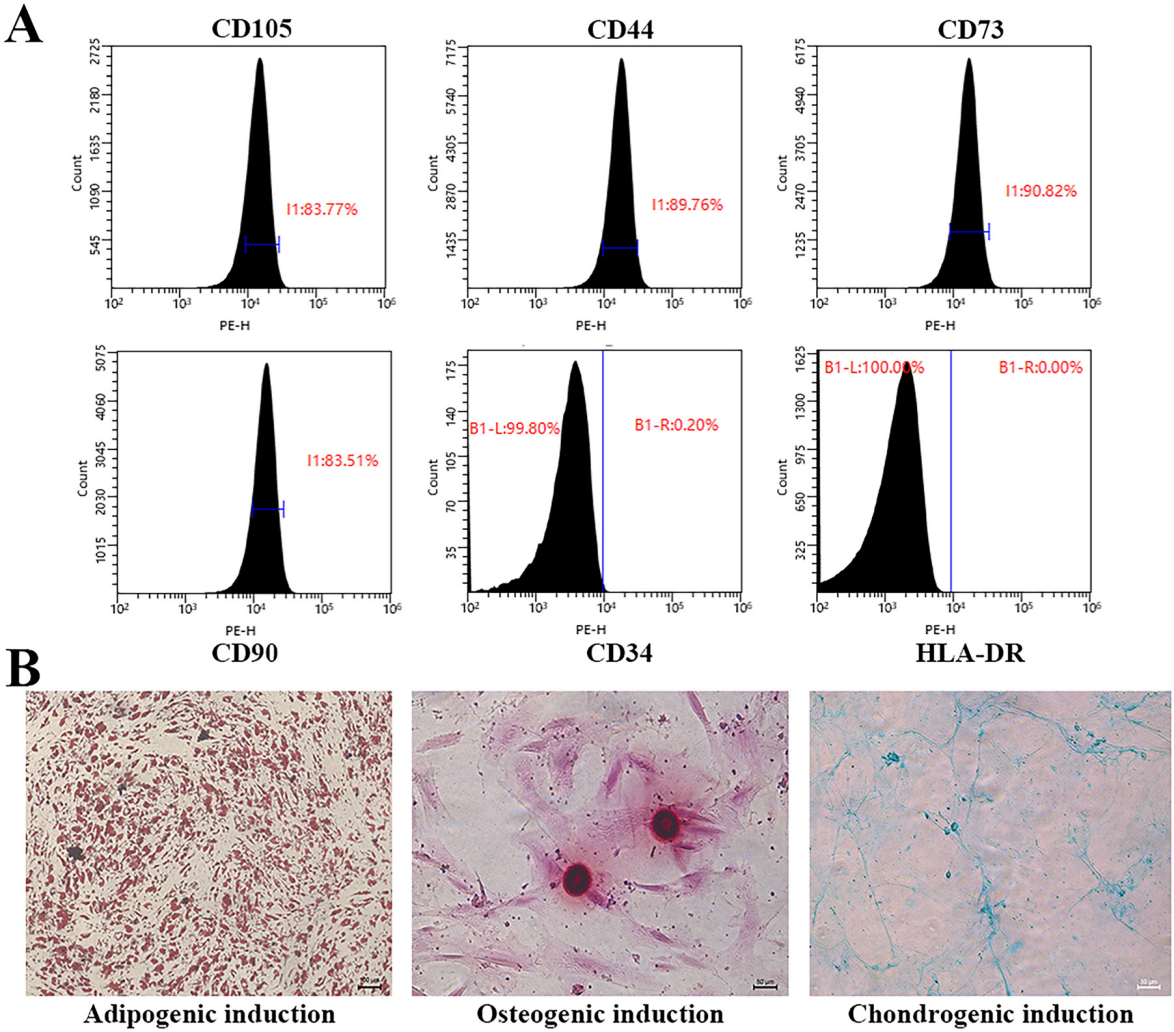
Expression of surface markers and results of trilineage differentiation in hUSCs.

From the experimental results, hUSCs demonstrated positive expression of CD105, CD44, CD73, and CD90, while CD34 and HLA-DR were negatively expressed (Figure 2A). These findings are consistent with the established immunophenotypic profile of mesenchymal stem cells.

### 3.3 Trilineage differentiation potential of hUSCs

Following 14 days of adipogenic induction, lipid droplets with a granular morphology were observed in the 6-well plate. These droplets appeared red upon Oil Red O staining, indicating the adipogenic differentiation potential of hUSCs. After 21 days of osteogenic induction, the hUSCs exhibited a morphological transition from a spindle-shaped to a flattened form, with increased cell density and overlapping growth. Alizarin Red staining indicated red calcified nodules, demonstrating osteogenic differentiation capacity. Additionally, after 21 days of chondrogenic induction, Alcian Blue staining identified mucopolysaccharide deposits external to some cells, appearing light blue in color, indicative of chondrogenic differentiation ability (Figure 2B). This inherent trilineage plasticity underscores the multipotent nature of hUSCs, enabling them not only to act as direct progenitors for urethral smooth muscle and urothelial cells but also to function as bioactive factories that secrete trophic factors – consistent with the exosome-mediated migration effects observed. Such differentiation competence likely underpins their reparative capacity within the porcine urethral matrix by creating a regenerative microenvironment that supports both cell recruitment and eventual tissue maturation.

### 3.4 Morphological characteristics, size, concentration, and protein expression of hUSCs-Exo

**Figure 3:**
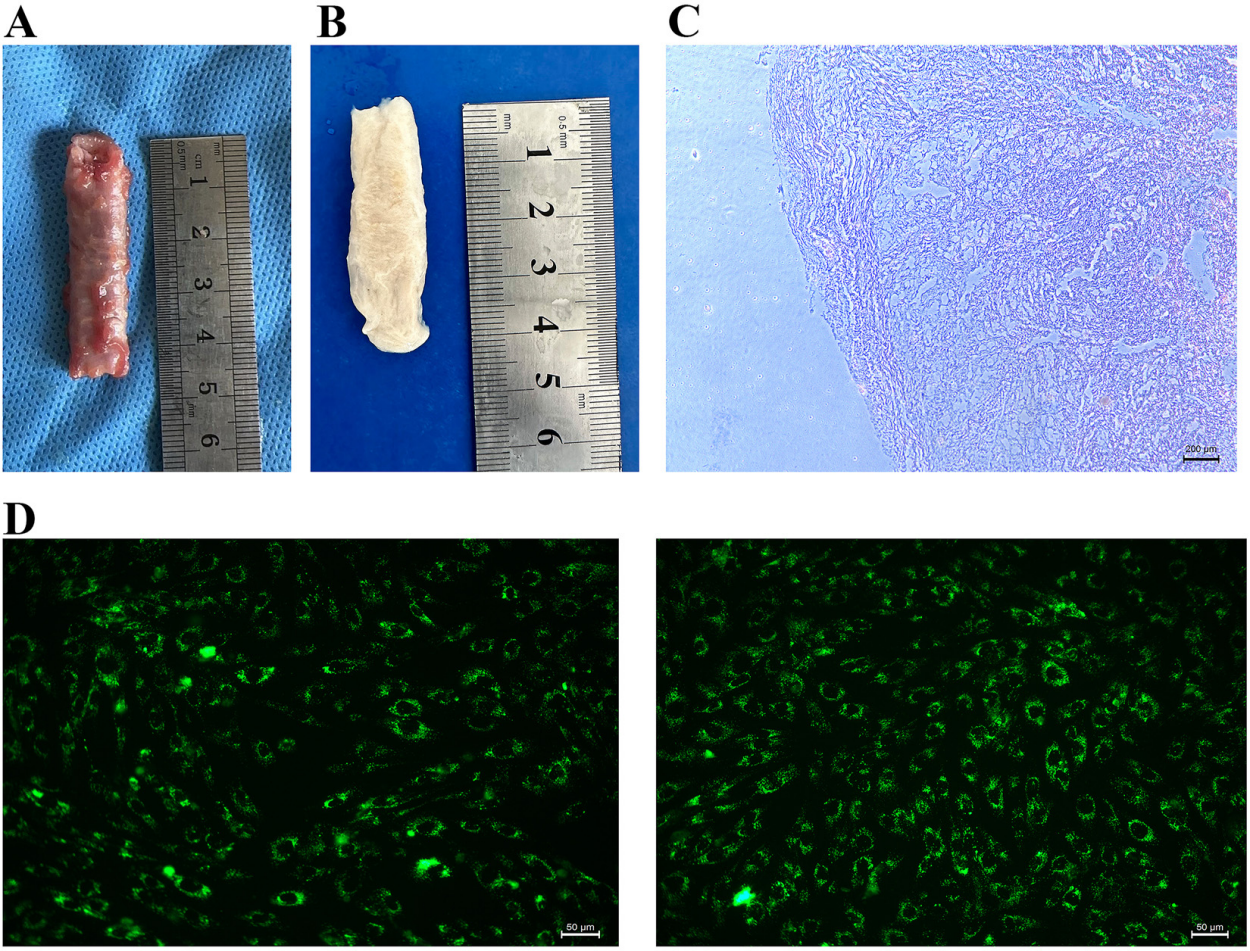
Characterization of porcine urethral decellularized matrix and Dio-labeled hUSCs. (A) Gross appearance of native porcine urethra. (B) Gross appearance of porcine urethral decellularized matrix. (C) H&E staining indicating fibrous structure devoid of cellular components. (D) Dio staining indicating green fluorescence of hUSCs on the matrix (200×).

Under transmission electron microscopy, hUSCs-Exo appeared as approximately round or oval vesicle-like structures (Figure 3A). BCA assay demonstrated that the protein concentration of the isolated exosomes was approximately 5 μg/μL.

### 3.5 Gross specimen and H&E staining of porcine urethral decellularized matrix

The pig urethra appeared light pink in color and measured approximately 4.5 cm in length (Figure 3A). The porcine urethral decellularized matrix exhibited a white gross appearance, characterized by a rough surface and visible villous fibers (Figure 3B). H&E staining of the decellularized matrix indicated the absence of cellular structures, with pink, strip-like fibrous components observed (Figure 3C).

### 3.6 Dio staining results of hUSCs

To enable real-time observation of the complexation between hUSCs and the porcine urethral decellularized matrix, cell membranes were stained with Dio. Under fluorescence inverted microscopy, the hUSCs exhibited an elliptical to round morphology, with the cell membranes distinctly stained green (Figure 3D). The stable membrane integration of Dio allows non-invasive, longitudinal tracking of hUSC progeny without compromising viability or exosome signaling, thereby providing a dynamic readout of how hUSCs-Exo modulate cell colonization kinetics within the scaffold’s three-dimensional architecture.

### 3.7 Cell scratch assay

**Figure 4:**
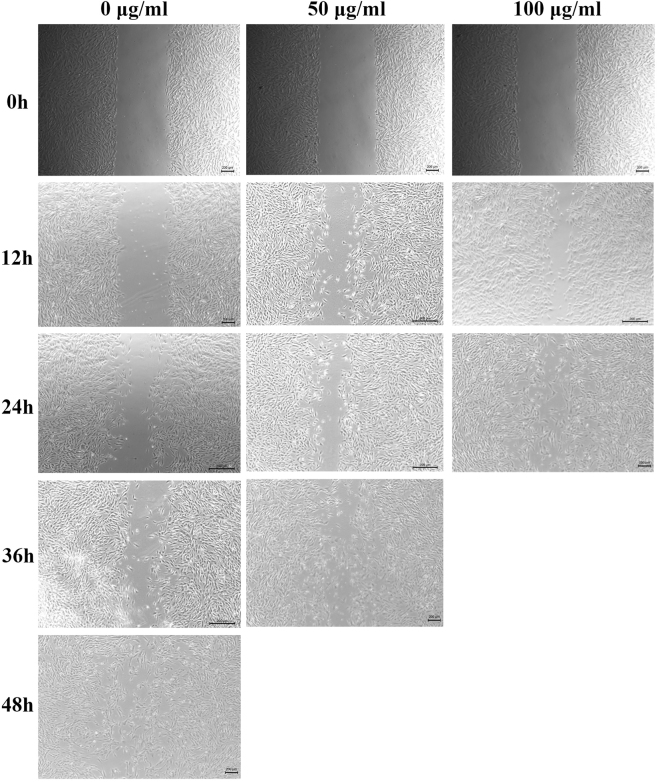
Comparison of scratch assay results among the groups treated with 0 μg/mL, 50 μg/mL, and 100 μg/mL hUSCs-Exo.

In the cell scratch assay, increased cell migration into the scratch area was observed at 12 h in both the 50 μg/mL and 100 μg/mL hUSCs-Exo groups compared to the 0 μg/mL group, with the highest level of migration noted in the 100 μg/mL group. By 24 h, the scratch area in the 100 μg/mL hUSCs-Exo group had nearly closed, whereas closure occurred at approximately 36 h in the 50 μg/mL group and around 48 h in the 0 μg/mL group. These findings indicate that both 50 μg/mL and 100 μg/mL concentrations of hUSCs-Exo promoted scratch healing in hUSCs, with the 100 μg/mL group demonstrating the most pronounced effect (Figure 4).

### 3.8 Transwell assay

**Figure 5:**
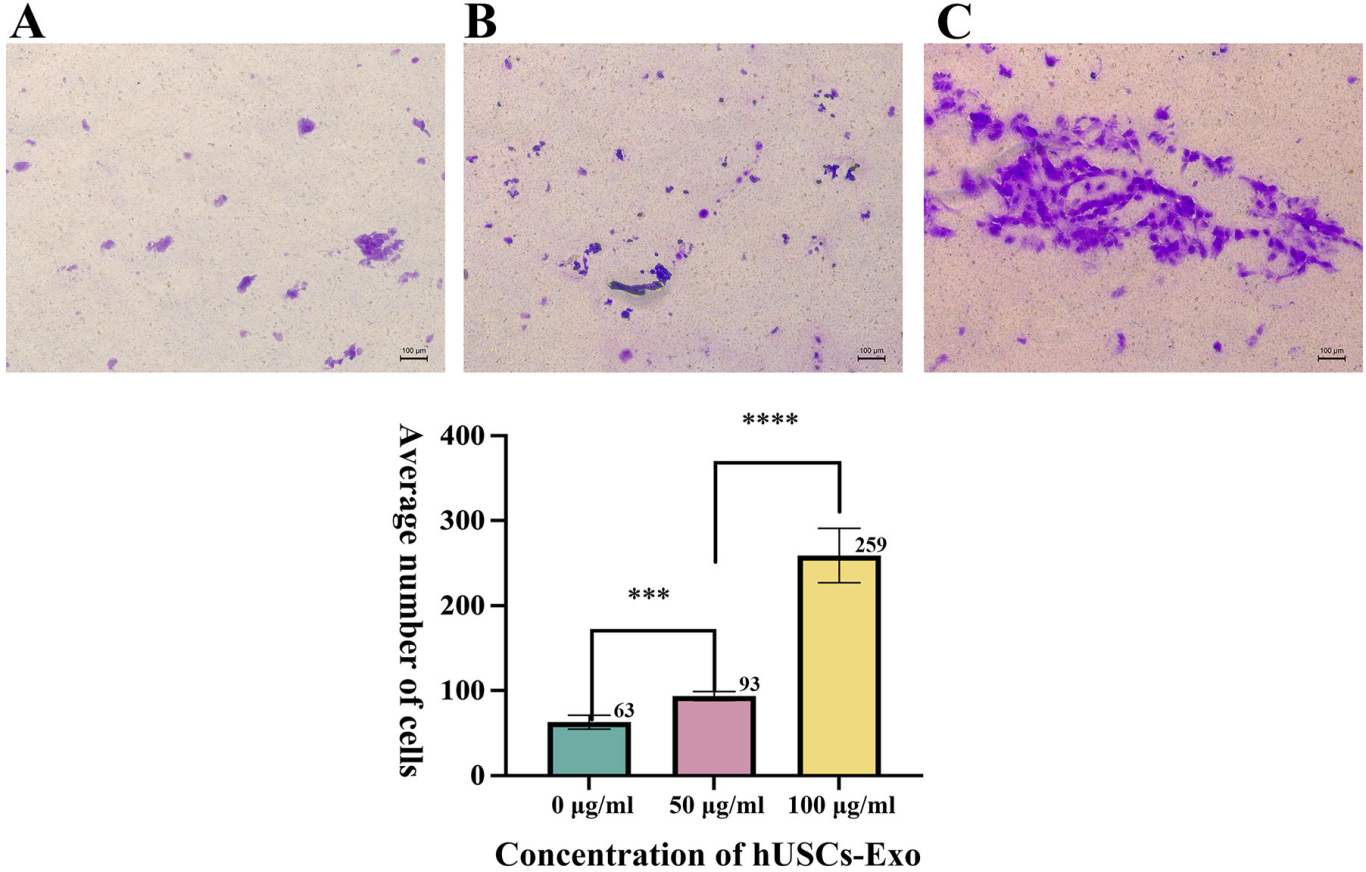
Effect of different concentrations of hUSCs-Exo on hUSC migration. (A–C) cell migration counts for 0 μg/mL, 50 μg/mL, and 100 μg/mL hUSCs-Exo groups, respectively (100×). Statistical significance indicated as ***, ****: *p* < 0.05.

Microscopic examination of the three Transwell chamber groups indicated distinct differences in cell migration counts. The cell migration count for the 0 μg/mL hUSCs-Exo group was (62.8 ± 8.17) cells; for the 50 μg/mL hUSCs-Exo group, it was (93.4 ± 5.27) cells; and for the 100 μg/mL hUSCs-Exo group, it was (259 ± 31.98) cells (Figure 5). Both the 50 μg/mL and 100 μg/mL hUSCs-Exo groups exhibited significantly higher cell migration compared to the 0 μg/mL group, with the 100 μg/mL group displaying the highest migration count. Statistical analysis indicated a significant difference among the three groups (*p* < 0.05) (Figure 5). This dose-dependent enhancement suggests that exosomal cargo, likely comprising microRNAs and growth factors, activates key signaling pathways that drive cytoskeletal rearrangement and chemotaxis in hUSCs.

### 3.9 Results of hUSCs and porcine urethral decellularized matrix complexation

At 24 h after seeding, fluorescence inverted microscopy revealed a small number of hUSCs distributed on the porcine urethral decellularized matrix in the control group. The cells exhibited an elliptical to round morphology and were irregularly arranged. No significant differences in cell distribution or attachment were observed among the groups treated with hUSCs-Exo at protein concentrations of 0, 50, and 100 μg/mL.

**Figure 6:**
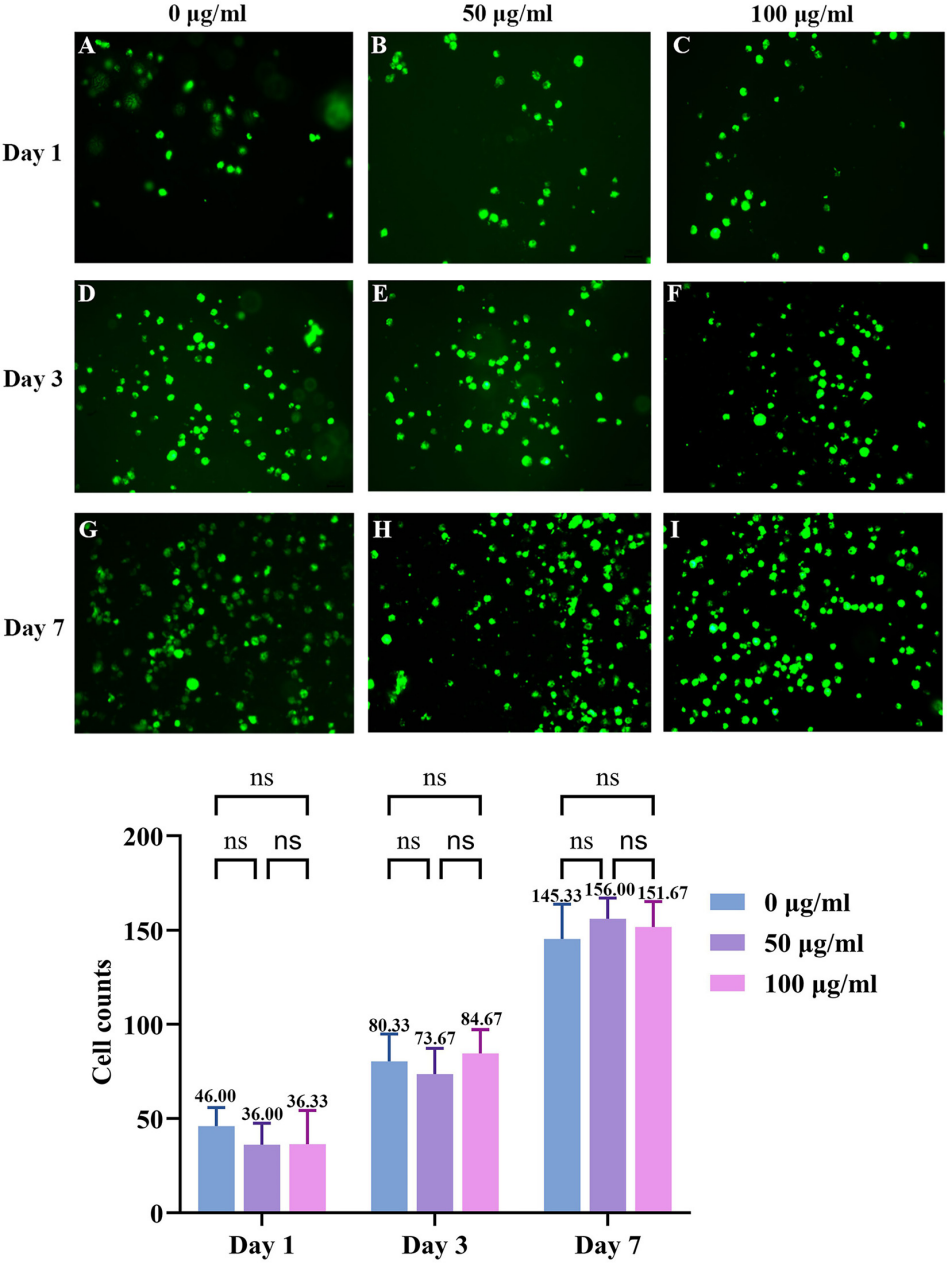
Fluorescence inverted microscopy observation of the complexation between hUSCs and porcine urethral decellularized matrix on days 1, 3, and 7 of co-culture.

Following 3 days of co-culture, cell numbers increased in all groups compared to earlier time points. Some cells had infiltrated the interior of the scaffold, resulting in blurred images at different focal planes. By day 7, cell numbers had increased markedly, and colony formation was evident in all groups. Cells were more densely distributed in thinner regions of the urethral scaffold and less abundant in thicker areas (Figure 6). Quantitative analysis of average cell counts across groups at different time points revealed no statistically significant differences (*p* > 0.05).

### 3.10 H&E staining and immunohistochemistry results of hUSCs and porcine urethral decellularized matrix complexation

**Figure 7:**
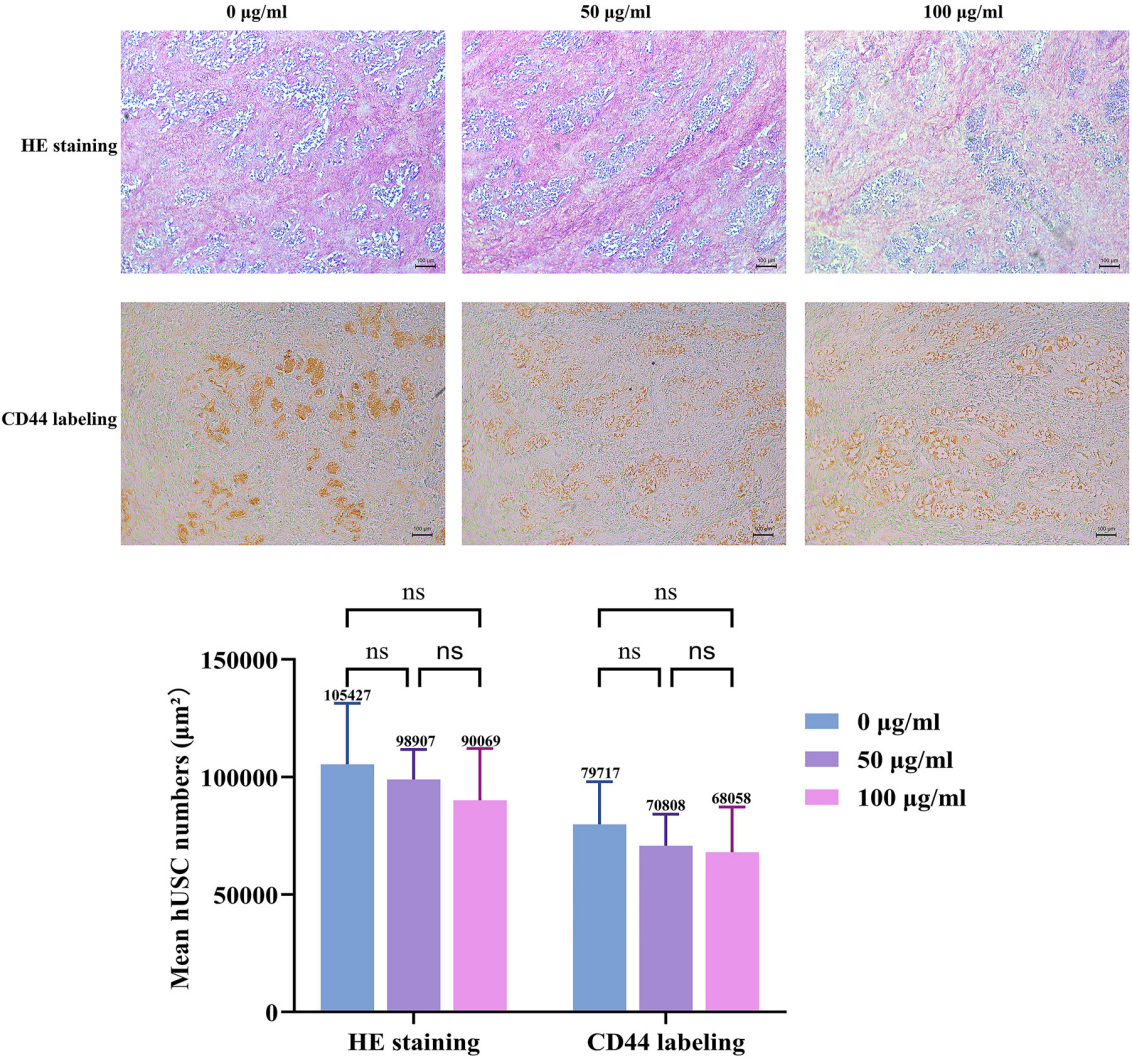
H&E and CD44 immunohistochemical staining of porcine urethral decellularized matrix seeded with hUSCs (100×), demonstrating cellular integration within the scaffold.

H&E staining conducted on day 7 of complexation demonstrated that, in all three groups (0 μg/mL, 50 μg/mL, and 100 μg/mL), the porcine urethral fibrous matrix appeared light pink, and the cell nuclei were stained bluish-purple. The hUSCs were identifiable within the fibrous scaffold, indicating successful cellular attachment to the matrix (Figure 7). Immunohistochemical staining targeting CD44, which is highly expressed on hUSC membranes, resulted in yellow staining, further confirming the presence and integration of hUSCs within the scaffold (Figure 7). The persistent CD44 positivity across all groups corroborates that hUSCs maintain their mesenchymal identity within the scaffold, yet the lack of exosome-dependent proliferation suggests that bioactive cues within the decellularized matrix may saturate the local microenvironment, rendering supplemental exosomes redundant for growth. Furthermore, the dense collagen network likely restricts exosome penetration to the scaffold’s deeper zones, thereby limiting their bioavailability to infiltrating cells and favoring migration-permissive rather than mitogenic responses.

## 4 Discussion

Recent advances in tissue engineering and regenerative medicine have enabled the development of novel biomaterials for urethral repair. A variety of graft materials, including fibrous scaffolds, decellularized matrices, and recellularized tissues, have been investigated for urethral reconstruction [4], 13]. Among these options, the porcine urethral decellularized matrix used in the present study offers several advantages. First, as naturally derived material, porcine urethra is cost-effective and readily available compared with many synthetic or allogenic biomaterials. Second, the genetic and anatomical features of the of porcine urethra closely resemble those of human urethra, making it a promising graft candidate for the treatment of long-segment urethral strictures.

In this study, male porcine urethra was harvested, decellularized using trypsin, and evaluated by H&E staining. The resulting scaffold exhibited a preserved fibrous architecture with no detectable residual cellular components.

Previous studies have highlighted the critical role of seed cells in enhancing urethral repair when combined with decellularized scaffolds [14]. The selection of seed cells is therefore a key determinant of tissue-engineered constructs. Mesenchymal stem cells, in particular, possess immunomodulatory properties that regulate inflammatory responses and tissue remodeling [15]. In this study, hUSCs isolated from healthy adult male donors were used as seed cells. These cells displayed phenotypic characteristics consistent with mesenchymal stem cells, in agreement with previous reports [16], 17].

The use of hUSCs as seed cells in combination with a porcine urethral decellularized matrix offers several advantages. hUSCs are easily and noninvasively obtained, exhibit multipotent differentiation potential, and represent a viable cell source for engineered urethral tissue. In addition, the use of autologous hUSCs may reduce the risk of immune rejection after implantation. Consequently, the incorporation of hUSCs into tissue-engineering strategies has become an area of growing interest. For example, Song et al. applied decellularized small intestinal submucosa seeded with hUSCs for bladder repair and regeneration [18]. Zhao et al. demonstrated that hUSCs could differentiate into urothelial and smooth muscle cell lineages and, when seeded onto a vascular extracellular matrix scaffold, could be used for ureteral reconstruction [8]. These studies support the feasibility of combining hUSCs with decellularized matrices for urinary tract tissue engineering.

It is now widely accepted that exosomes are a major mechanism through which mesenchymal stem cells exert their biological effects [19], 20]. Exosomes have been shown to promote tissue repair, suppress inflammatory responses, and enhance angiogenesis, among other functions [21], [22], [23]. In this study, hUSCs-Exo were isolated from culture supernatants using an exosome extraction reagent followed by centrifugation. Transmission electron microscopy revealed vesicle-like particles with an oval morphology, and nanoparticle tracking analysis showed a mean particle diameter of approximately 128 nm. These characteristics are consistent with previously reported exosomal profiles, confirming the successful isolation of exosomes [24], [25], [26].

Prior studies have indicated that exosomes can enhance both horizontal and vertical migration of various cell types [27], 28]. For instance, Tong et al. reported that both CD133^+^ hUSC-derived exosomes and unselected hUSCs-Exo enhanced the *in vitro* migration of bone marrow mesenchymal stem cells during co-culture [29]. Consistent with these findings, this study utilized cell scratch and transwell assays to confirm that hUSCs-Exo promoted horizontal and vertical migration of hUSCs. While earlier work examined either exosomes or scaffolds separately, our study is the first to integrate human urine-derived stem cell exosomes (hUSCs-Exo) with a porcine urethral decellularized matrix into a single tissue-engineering platform and to quantify their combined effects on cell migration, spatial distribution, and proliferation within the same scaffold. Using real-time fluorescent tracking and immunohistochemical quantification, we provide a reproducible *in-vitro* paradigm for evaluating cell–scaffold crosstalk. The finding that exosomes selectively enhance migration without affecting proliferation offers a clear baseline for future mechanistic studies and guides rational dose/design optimization before *in-vivo* translation.

To investigate if hUSCs-Exo influence the interaction between hUSCs and the porcine urethral decellularized matrix, co-culture experiments were conducted using Dio-stained hUSCs. The results demonstrated that hUSCs remained viable and proliferative when cultured on trypsin-treated porcine urethral decellularized matrix. However, following the addition of hUSCs-Exo, no significant differences in cell survival or proliferation were observed when compared to the control group. These findings suggest that hUSCs-Exo may not exert a significant effect on cell proliferation within this composite system.

Several factors may account for this outcome: (1) hUSCs are known to continuously secrete exosomes endogenously, and as cell numbers increase, the concentration of hUSCs-Exo may not remain a limiting factor in later culture stages; (2) the simultaneous co-culture of hUSCs, hUSCs-Exo, and porcine urethral decellularized matrix may influence exosomal function, potentially through interactions with the scaffold material. Additionally, (3) the paracrine effects of hUSC-Exo were evaluated exclusively on hUSCs themselves, without assessing their impact on primary urethral cell types – urothelial, smooth muscle, and endothelial cells (EC) – that are essential for functional tissue reconstruction and vascularization. These possibilities warrant further investigation in future studies aimed at optimizing exosome delivery and dissecting their orchestrating roles across multiple urethral lineages.

In addition, this study did not incorporate additional control conditions such as VEGF-supplemented constructs or acellular scaffolds. Because the experimental design focused on comparing hUSC responses to different concentrations of hUSC-derived exosomes within a consistent scaffold–cell environment, all groups were constructed using identical seeded matrices. Future studies, particularly those involving *in vivo* models or multiple urethral cell types, will benefit from including such control conditions to more fully delineate scaffold-dependent and exosome-independent effects.

## 5 Conclusions

In conclusion, this study establishes human urine-derived stem cells as robust seed cells for urethral tissue engineering, demonstrating their sustained viability, proliferative activity, and multipotent differentiation potential within a porcine decellularized matrix. The central finding reveals that hUSC-derived exosomes selectively enhance hUSC migration in a concentration-dependent manner, yet exert no significant effect on cellular proliferation or scaffold colonization kinetics during the 7-day culture window. This dichotomy suggests that the scaffold microenvironment may either saturate proliferative signaling pathways or limit exosome bioavailability in deeper regions. Our work provides a functionally validated, *in-vitro* platform for exosome-mediated urethral regeneration and underscores the necessity of optimizing exosome delivery strategies to fully realize their therapeutic potential in scaffold-based applications.
